# Opposite temperature effect on transport activity of KCC2/KCC4 and N(K)CCs in HEK-293 cells

**DOI:** 10.1186/1756-0500-4-526

**Published:** 2011-12-09

**Authors:** Anna-Maria Hartmann, Hans Gerd Nothwang

**Affiliations:** 1 Department of Neurogenetics, Institute for Biology and Environmental Sciences, Carl von Ossietzky University, Carl von Ossietzky Straße 9-11, 26129 Oldenburg, Germany; 2 Research Center Neurosensory Science, University of Oldenburg, 26129 Oldenburg, Germany

## Abstract

**Background:**

Cation chloride cotransporters play essential roles in many physiological processes such as volume regulation, transepithelial salt transport and setting the intracellular chloride concentration in neurons. They consist mainly of the inward transporters NCC, NKCC1, and NKCC2, and the outward transporters KCC1 to KCC4. To gain insight into regulatory and structure-function relationships, precise determination of their activity is required. Frequently, these analyses are performed in HEK-293 cells. Recently the activity of the inward transporters NKCC1 and NCC was shown to increase with temperature in these cells. However, the temperature effect on KCCs remains largely unknown.

**Findings:**

Here, we determined the temperature effect on KCC2 and KCC4 transport activity in HEK-293 cells. Both transporters demonstrated significantly higher transport activity (2.5 fold for KCC2 and 3.3 fold for KCC4) after pre-incubation at room temperature compared to 37°C.

**Conclusions:**

These data identify a reciprocal temperature dependence of cation chloride inward and outward cotransporters in HEK-293 cells. Thus, lower temperature should be used for functional characterization of KCC2 and KCC4 and higher temperatures for N(K)CCs in heterologous mammalian expression systems. Furthermore, if this reciprocal effect also applies to neurons, the action of inhibitory neurotransmitters might be more affected by changes in temperature than previously thought.

## Background

Cation chloride cotransporters (CCCs) are pivotal plasma membrane proteins for many physiological processes such as transepithelial salt transport, neuronal chloride homeostasis, and cell volume regulation [1,2]. Due to their essential functions, mutations of these transporters are associated with a variety of disorders such as deafness, renal dysfunction, seizures, and chronic pain, or are not compatible with life [1,2]. The mammalian genome codes for nine family members, seven of which are Cl^-^-transporters. The Na^+^-driven NCC, NKCC1, and NKCC2 are Cl^-^-inward transporters, whereas the K^+^-driven family members KCC1 to KCC4 represent Cl^-^-outward transporters [1]. Due to their opposite transport direction, various mechanisms of reciprocal regulation of NKCCs and KCCs have been reported. This includes opposite effects of phosphorylation, interaction partners such as WNK [3] and CIP1 [4,5], or membrane rafts on the transport activity [4,6].

The vital importance of CCCs has resulted in a high interest in regulatory mechanisms and structure-function relationships. Most studies addressing these issues have been performed in heterologous expression systems. HEK-293 cells represent the preferred mammalian expression system [4-10]. As a high transport activity is a prerequisite for functional studies, it is important to optimize the parameters in this cell line for functional analyses. A recent analysis of NKCCs in HEK-293 identified a marked increase in transport activity when shifting the cell line from room temperature (RT) to 37°C [11]. This observation is in agreement with previous NKCC flux measurements in red cells [12]. Furthermore, analyses of the K^+^-transport in red cells, which is likely mediated by KCC1 and KCC3 [13], revealed also a higher flux at elevated temperatures [14].

In light of these temperature dependent transport activity of various CCC family members, we investigated the temperature effect on KCC2 and KCC4 transport activity in HEK-293 cells. KCC2 is a neuronal isoform which is active under isotonic conditions [7] and localized in non-membrane rafts [6], whereas KCC4 resides in membrane rafts [15] and is active under hypotonic conditions [16]. Both transporters form one branch of KCCs, whereas the other one is formed by KCC1 and KCC3 [1]. The activity of both transporters was significantly higher after a short preincubation at RT, contrasting the previously reported temperature effects on CCCs.

## Result

**Figure 1:**
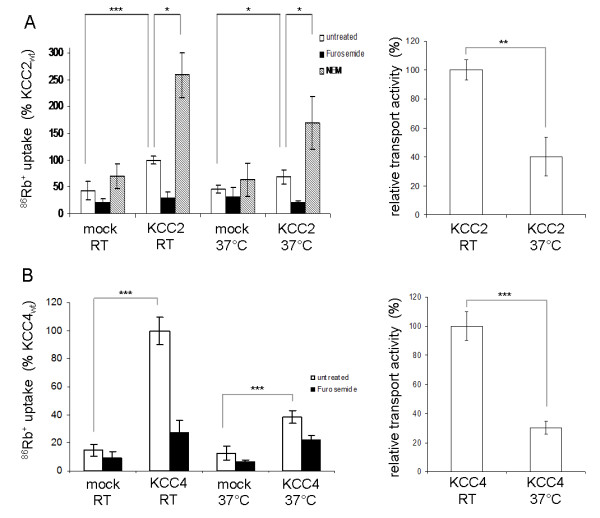
**Temperature effect on KCC2 and KCC4 in HEK-293 cells**. HEK-293 cells were transfected with KCC2 (**a**) and KCC4 (**b**). Before ^86^Rb^+ ^uptake cells were incubated for 30 min in preincubation buffer at room temperature or 37°C. An empty vector was used for mock transfection. Both KCC2 (*p *= 0.004) and KCC4 (*p *= 3.42 × 10^-5^) showed significant increase in transport activity after preincubation at room temperature compared to 37°C (left handed part). Relative transport activity was calculated after background subtraction (right handed part). All ^86^Rb^+ ^uptakes were sensitive to 2 mM furosemide. KCC2 transport activity could be significantly stimulated by 1 mM NEM. ***, *p *< 0.001; **, *p *< 0.005; *, *p *< 0.05.

Temperature-dependence of KCC2 and KCC4 transport activity in HEK-293 cells was determined after transient transfection by ^86^Rb^+ ^flux measurements. All ^86^Rb^+ ^-uptake was sensitive to the KCC inhibitor furosemide (Figure 1). KCC2 transfected cells displayed a significant 1.5 fold increase in ^86^Rb^+ ^uptake compared to mock-transfected control cells (Figure 1a, left part), when preincubated at 37°C prior flux measurements. When preincubated at RT for 30 min, the activity was 2.3 fold increased compared to mock-transfected cells. Importantly, the transport activity of KCC2 at RT was 2.5 fold increased compared to its activity at 37°C after background subtraction (Figure 1a, right part). This difference was highly significant (*p *= 0.004). To investigate whether KCC2 was still capable of a high transport activity at 37°C, we stimulated its activity by different agents. Treatment with NEM, a known activator of KCC2 [17], resulted in a ~ 2.5 fold increased ^86^Rb^+ ^uptake, which was similar to the 2.6 fold stimulation observed at RT (Figure 1a). In addition, treatment with 7.5 μM staurosporine, a protein kinase inhibitor, resulted in an 11 fold increase in KCC2 mediated ^86^Rb^+ ^uptake (data not shown). These data demonstrate that KCC2 is still able of a high transport activity after preincubation at 37°C.

KCC4 transfected cells displayed a significant 3 fold increase in ^86^Rb^+^-uptake at 37°C compared to mock-transfected cells (Figure 1b, left part). Similar to KCC2, preincubation at RT considerably increased the difference in the ^86^Rb^+^-uptake between KCC4 transfected and mock transfected cells. We observed a 6.7 fold increase in ^86^Rb^+^-flux in KCC4 expressing cells. After background subtraction, KCC4 had a significant 3.3 fold higher activity at RT compared to preincubation at 37°C (*p *= 3.4 × 10^-5^, right part). Taken together these data demonstrate a strong temperature-dependency of KCC2 and KCC4 transport activity in HEK-293 cells.

## Discussion

The main finding of this study is that transport activity of KCC2 and KCC4 is significantly higher after 30 min preincubation at RT compared to 37°C. The observed fold changes of 2.5 for KCC2 and 3.3 for KCC4 were in a similar range. This inverse correlation with temperature contrasts the increase in transport activity reported for NKCC1 and NKCC2 at 37°C compared to RT in the same heterologous expression system [11]. These data imply that 30-60 min preincubation at RT is the preferred condition for the analysis of KCC2/KCC4 and 37°C for NKCCs in the widely used HEK-293 cells in order to obtain high transport activity.

Whether our findings for KCC2 and KCC4 can be extended to the other branch of KCCs, i.e. KCC1 and KCC3 remains to be elucidated. Previous studies in red cells have indicated that KCC1 and KCC3 activity increased with elevated temperatures [13,14]. This opposite effect could either reflect differences in the two KCC branches or a cell-type specific modulation of KCCs by temperature. To answer this issue requires insight into the underlying mechanism. Both for KCCs and NKCCs, the effect occurs within 30-60 min and various scenarios could be envisaged. i) A temperature-dependent modification such as phosphorylation might occur [11]. An increased activity of the known CCC regulators WNK and SPAK at 37°C would result in an increase in the transport activity of NKCCs and a decrease in activity of KCCs [18,19]. Both WNK1 and SPAK are present in HEK-293 cells [20]. It might therefore be worthwhile to investigate the contribution of these kinases on the observed temperature effects. ii) The temperature might affect folding and trafficking of newly synthesized transporters, their structural configuration in the plasma membrane, or reduce endocytosis of plasma membrane residing transporters [21]. These mechanisms, however, should be rather independent of the cell-type analysed. iii) CCCs are secondary active transporters which depend on the Na^+^/K^+^-ATPase [1]. Therefore, altered activity of this pump would influence CCC-mediated transport. Indeed, ablation of the Na^+^/K^+^-ATPase subunit Atp1a2 results in increased neuronal [Cl]_i _[22]. Elevated preincubation temperatures in buffers containing no nutrients might result in a compromised metabolic state of the cell, resulting in decreased Na^+^/K^+^-ATPase activity. However, we consider this explanation unlikely as altered activity of Na^+^/K^+^-ATPase would influence KCCs and N(K)CCs in the same direction. We also blocked the Na^+^/K^+^-ATPase throughout the experiments by adding ouabain prior exposure to different temperatures. In addition, the observed increase of NKCC1 activity after a 60 min incubation at 37°C compared to RT [11] strongly argues against a compromised metabolism in HEK-293 cells after preincubation for up to 1 h at elevated temperatures. In line with this, our data demonstrate that KCC2 activity can be still strongly stimulated by staurosporine or NEM after preincubation at 37°C.

The observed temperature effect might have important biomedical implications for the analyses of inhibitory neurotransmission in neurons. The action of the two major inhibitory neurotransmitters GABA and glycine in neurons depends on the intracellular chloride concentration [Cl]_i _[2,23], as their receptors represent ligand-gated chloride channels. A high [Cl]_i _hence results in depolarization, whereas low [Cl]_i _causes hyperpolarization. Since the electrochemical equilibrium for chloride is close to the resting membrane potential, subtle reciprocal changes in the transport activity of NKCC1 and KCC2 and consequently in [Cl]_i _can inverse the action of these neurotransmitters.

## Conclusion

In summary, our data demonstrate that KCC2 and KCC4 transport activity is strongly influenced by temperature in heterologous mammalian expression systems. A short preincubation (30 min) at room temperature is therefore recommended to obtain high transport activity. In addition, the opposite effect of temperature on KCC2/KCC4 and NKCCs adds another facet to the reciprocal regulation of these two branches of the cation chloride cotransporter gene family. Futures studies have to address whether the temperature sensitivity holds true for in vivo analyses as well.

## Materials and methods

### Plasmid constructs

We used previously reported pCDNA3.1 expression vectors with the open reading frames of rat KCC2b (GenBank accession no. NM_134363) and mouse KCC4 (GenBank accession no. NM_011390) [8].

### Determination of K^+^-Cl^- ^cotransport

Transport activity was determined by measuring uptake of ^86^Rb^+ ^(PerkinElmer Life Sciences) in HEK-293 cells. Cells were cultured in Dulbecco's Modified Eagle Medium (Invitrogen, Darmstadt, Germany) and transfected using TurboFect (Fermentas, St. Leon-Roth, Germany). Cells were harvested 48 h after transfection and transferred into poly-L-lysine-coated wells of a 6-well-culture dish and incubated for 3 h. After removal of the medium, cells were incubated in 1 ml preincubation buffer (100 mM N-methyl-D-glucamine-chloride, 5 mM KCl, 2 mM CaCl_2_, 0.8 mM MgSO_4_, 5 mM glucose, 5 mM HEPES, pH 7.4, 0.1 mM oubain) for 30 min at RT or 37°C. Treatment with 7.5 μM staurosporine for 30 min, 1 mM NEM or 2 mM fouresemide for 15 min occurred in the preincubation buffer. A 10 min uptake period followed in preincubation buffer supplemented with 1 μCi/ml ^86^Rb^+ ^at RT. At the end of the uptake period, cells were washed three times in 1 ml ice-cold preincubation buffer to remove extracellular tracer. Cells were lysed in 500 μl 0.25 M NaOH for 1 h and then neutralized with 250 μl pure acetic acid. ^86^Rb^+ ^uptake was assayed by Cerenkov radiation, and the protein amount was determined by BCA (Thermo Fisher Scientific, Bonn, Germany). In addition, expression of the respective construct was determined for each flux measurement by immunoblot analysis. Two biological and three technical replicas were performed for each experiment. Data are given as mean ± standard error deviation. Significant differences between the groups were analyzed by a Student's *t*-test.

## Competing interests

The authors declare that they have no competing interests.

## Authors' contributions

AMH carried out the experiments and wrote the manuscript. AMH and HGN designed the experiments. HGN helped to draft the manuscript. Both authors read and approved the final manuscript.
